# Récidive à localisation rare d'une synovite villo-nodulaire du genou

**DOI:** 10.11604/pamj.2016.23.164.8763

**Published:** 2016-04-06

**Authors:** Mohamed Amine Karabila, Hmouri Ismail

**Affiliations:** 1 Service de Chirurgie Traumato-orthopédie, CHU Ibn Sina, Rabat, Maroc

**Keywords:** Récidive, synovite, genou, Recurrence, synovitis, knee

## Image en médecine

La synovite villo-nodulaire est une affection bénigne rare, caractérisée par une hyperplasie villeuse ou nodulaire de la synoviale d’étiologie inconnue. Elle se présente par des douleurs et une tuméfaction articulaire. L'IRM est l'examen radiologique de choix en cas de suspicion clinique, mais l'examen histopathologique donne le diagnostic définitif. L’évolution naturelle est marquée par un haut taux de récidives et le traitement est donc souvent difficile. Nous présentons le cas d'un patient âgé de 30 ans présentant une récidive d'une synovite villo-nodulaire à localisation postérieure du genou (A, B, C) gauche après une synovectomie sub-totale en 2012. Pour le deuxième geste, le patient a bénéficié d'une synovectomie postérieure (D) par voie postéro interne après repérage du pédicule poplité. Le traitement de cette pathologie est mal codifiée vu la rareté de l'affection, mais la synovectomie totale reste la meilleure option thérapeutique pour diminuer le risque de récidive

**Figure 1:**
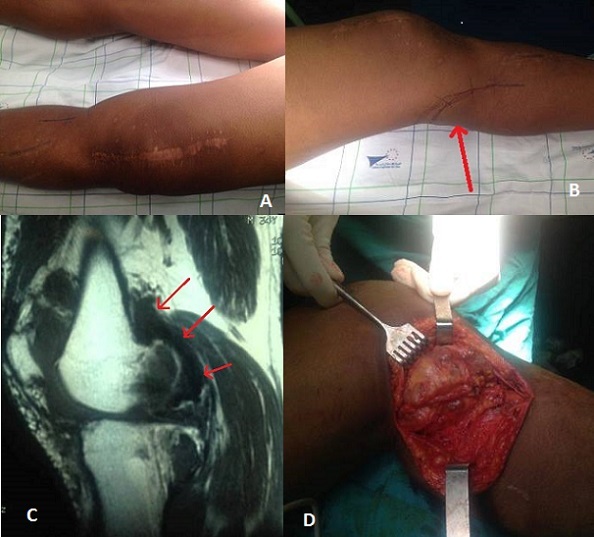
A) aspect tuméfié du genou avec une cicatrice médiane en rapport avec la synovectomie réalisée en 2012; B) préparation du trajet de l'incision en regard de la tuméfaction postérieure du genou; C) IRM du genou montrant la volumineuse masse en hypo-signal en T2; D) aspect peropératoire de la synovite

